# Efficacy of Endometrial Cancer Follow-up Protocols: Time to Change?

**DOI:** 10.1055/s-0040-1721352

**Published:** 2021-01-29

**Authors:** Amina Lubrano, Virginia Benito, Beatriz Pinar, Fernando Molano, Laureano Leon

**Affiliations:** 1 Department of Gynecology and Obstetrics, Complejo Hospitalario Universitario Insular Materno-Infantil, Las Palmas de Gran Canaria, Spain; 2 Depatment of Radiotherapy, Hospital Dr. Negrin, Las Palmas de Gran Canaria, Spain; 3 Department of Medical Oncology, Complejo Hospitalario Universitario Insular Materno-Infantil, Las Palmas de Gran Canaria, Spain; 4 Department of Pathology, Complejo Hospitalario Universitario Insular Materno-Infantil, Las Palmas de Gran Canaria, Spain

**Keywords:** endometrial cancer relapse, follow-up, survival

## Abstract

**Objective** The aim of the present study was to analyze relapse rates and patterns in patients with endometrial cancer with the aim of evaluating the effectiveness of current follow-up procedures in terms of patient survival, as well as the convenience of modifying the surveillance strategy.

**Methods** Retrospective descriptive study including all patients diagnosed with endometrial cancer relapse at the Department of Gynecology and Obstetrics of the Complejo Hospitalario Insular-Materno Infantil de Canarias, between 2005 and 2014.

**Results** Recurrence was observed in 81 patients (10.04% of the sample); 66.7% of them suffered relapse within 2 years and 80.2% within 3 years after the termination of the primary treatment; 41.9% showed distant metastases while the rest corresponded to local-regional (40.7%) or ganglionar (17.4%) relapse; 42% of these were symptomatic; 14 patients showed more than 1 site of relapse. Relapse was detected mainly through symptoms and physical examination findings (54.3%), followed by elevated serum marker levels (29.6%), computed tomography (CT) images (9.9%) and abnormal vaginal cytology findings (6.2%). No differences in global survival were found between patients with symptomatic or asymptomatic relapse.

**Conclusion** Taking into account that the recurrence rate of endometrial cancer is low, that relapse occurs mainly within the first 3 years post-treatment and that symptom evaluation and physical examination are the most effective follow-up methods, we postulate that a modification of the current model of hospital follow-up should be considered.

## Introduction

The incidence of cancer in Spain is rising. According to recent data from the Spanish Society of Medical Oncology, 277,394 new cases were diagnosed in 2019, and this figure is expected to increase to ∼ 315,413 new cases in 2035. 1 Such an increase in the number of cases along with lower mortality rates due to scientific advances in cancer screening, diagnosis and treatment, lead to higher prevalence rates and, consequently, to more survivors. From tumors affecting women, breast, colorectal and endometrial are most frequently associated with long-term survival. Endometrial cancer is the most frequent malignancy of the female genital tract in Western countries. About 382,069 new cases were diagnosed in 2018 worldwide. 2 In Spain, 6,784 new cases were detected in 2018. Survival is known to be closely related to disease stage, with local disease associated with > 95% survival at 5 years. 3 Given that endometrial cancer is the most common gynecological neoplasm and that most patients present with early-stage disease, the amount of patients to be followed-up after treatment is rather high. Traditionally, the objective of post-treatment follow-up protocols has been to diagnose possible treatment-associated complications and detect relapse as soon as possible. Currently, other goals related to long-term follow-up are emerging, for example, control of delayed toxicity, management of physical consequences, rehabilitation and promotion of health and healthy habits. Overall, the goal is to improve the quality of life of survivors, as well as to help them retake their social, familial and working life. 4

However, the follow-up model currently used for such patients, mainly focused on diagnosing relapse, is based on a multidisciplinary hospital-based approach, which may be redundant and requires several visits and interventions that lead to overuse of hospital resources. Thus, the aim of the present study was to analyze relapse rates and patterns in endometrial cancer patients managed at our hospital, to assess the effectiveness of current follow-up procedures in terms of patient survival, as well as the convenience of modifying the surveillance strategy.

## Methods

The present retrospective descriptive study included all patients diagnosed with endometrial cancer relapse at the Department of Gynecology and Obstetrics of the Complejo Hospital Universitario Insular Materno Infantil de Canarias, between 2005 and 2014.

In our center, surgery is the primary treatment for endometrial cancer. Based on the national and international guidelines in use during the considered period, we conducted complete hysterectomy with bilateral salpingo-oophorectomy plus cytology of peritoneal washing fluids. In patients with endometrioid adenocarcinoma of histological grades 1 or 2, an intraoperatory finding of myometrial involvement (invasion > 50%) leads to pelvic and paraaortic lymphadenectomy. In case of endometrioid adenocarcinoma grade 3 or histological type 2 (serous, clear cells, undifferentiated or carcinosarcoma), pelvic and paraaortic lymphadenectomy was performed regardless of myometrial involvement.

Traditionally, we have conducted surgery for endometrial cancer through laparotomy. However, since 2006, we adopted a minimally invasive approach, changing from 8% of laparoscopic surgeries in 2006 to > 95% in 2009.

The patients were staged by using the system of the International Federation of Gynecology and Obstetrics (FIGO) from 2009. A multidisciplinary committee for tumors (meeting weekly) evaluated and classified the patients into three risk-groups on the basis of their anatomic pathology outcomes: 1) low-risk: including tumors of endometrioid histology stage I, grade 1–2, < 50% myometrial invasion and negative lymphovascular space invasion (LVSI); 2) medium-risk: including tumors of endometrioid histology stage I, grade 1–2, ≥ 50% myometrial invasion and negative LVSI; and 3) high-risk: including endometrioid tumors grade 3, ≥ 50% myometrial invasion, stage II-III and type-2 endometrial cancer. Patients in the medium- or high-risk groups received adjuvant radiotherapy and/or chemotherapy treatment.

The follow-up protocol in the studied period included physical examination, cytology of the vaginal vault, measurement of tumor markers (CA 125) and imaging tests depending on the symptoms and findings. In-hospital oncologic follow-up visits took place every 4 months during the first 2 years post-treatment, every 6 months between the 2 ^nd^ and 5 ^th^ years post-treatment and once a year afterwards.

Relapse was defined as detection of disease after a 6-month disease-free period, following the termination of the primary treatment. Cases of relapse were evaluated taking into account: primary treatment, ganglionar involvement, disease stage, histological grade, symptoms, site of relapse, and the way of detection of relapse. Depending on the first relapse site, patients were grouped into local-regional relapse (vaginal vault, isolated pelvic relapse or peritoneal carcinomatosis), ganglionar relapse (pelvic and/or aortic) or systemic relapse (metastases of the bone, liver, lung, brain or supradiaphragmatic ganglia).

The cutoff point for the survival analysis was December 31 ^st^ , 2017. The present study was approved by the local Ethics Committee (protocol number 2020–034–1). The statistical analysis was conducted with the SPSS for Windows, version 12 (SPSS Inc., Chicago, IL, USA). Quantitative variables were compared by using the Student *t* -test or the Mann-Whitney test. Categorical variables were analyzed with the chi-squared test or the Fisher exact test. Survival rates were analyzed with the Kaplan-Meier technique. The Cox proportional hazard model was used to identify risk factors for relapse or survival. Statistical significance was considered for p-values < 0.05.

## Results

During the studied period, 806 patients were diagnosed with endometrial cancer (mean: 80.6 cases/year). The mean follow-up time was 42.94 months (standard deviation [SD] 30.3). Relapse was detected in 81 patients (10.04% of the sample). At the moment of diagnosis, these patients were 67.72 years old (range 41–96 years old) on average; and the FIGO classification of the initial tumor was: stage I in 41 patients (50.6%), stage II in 12 patients (14.8%), stage III in 22 patients (27.2%) and stage IV in 6 patients (7.4%); 70.3% (57 patients) had type-1 endometrial adenocarcinoma (endometrioid) and 29.7% (24 patients) had type-2 endometrial adenocarcinoma (nonendometrioid); 28.4% (23 patients) had been initially treated with surgery only, while the rest of them had received adjuvant treatment depending on their risk group. The average time to relapse was 23.86 months (SD 19.4). Table 1 shows the characteristics of the patients.

**Table 1:** Characteristics of patients with relapse

Characteristics	*n* = 81	Percentage (%)
FIGO 2009 stage		
I	41	50.6
II	12	14.8
III	22	27.2
IV	6	7.4
Histology		
Type 1	57	70.3
Type 2	24	29.7
Differentiation grade		
G1	24	29.6
G2	23	28.4
G3	34	42
Myometrial involvement		
< 0–50%	39	48.1
50%	38	46.9
No entry	4	5
Lymphadenectomy		
No	46	56.8
Yes	35	43.2
Tumor size		
< 4 cm	40	49.4
> 4 cm	33	40.7
Unknown	8	9.9
Lymphovascular involvement		
No	43	53
Yes	29	35.8
Unknown	9	11.2
Adjuvant treatment		
No	23	28.4
Radiotherapy (RT)	47	58
Chemotherapy/Hormone therapy	4	5
Radiotherapy + Chemotherapy	7	8.6
Relapse		
Local-regional	33	40.7%
Ganglionar	14	17.3%
Distant	34	41.9%
Multisite	14	17.3%

Within patients with relapse, 41.9% showed distant metastases, 40.7% showed local-regional, and 17.4% ganglionar relapse; 14 patients showed > 1 site of relapse (17.3%); 8.9% of recurrence cases (8 patients) belonged to the low-risk group, 14.8% (12 patients) to the medium-risk group, and 73.3% (61 patients) to high-risk groups; 66.7% of the relapses (54 cases) occurred within the 1 ^st^ 2 years post-treatment, and 80.2% (65 cases) within the 1 ^st^ 3 years post-treatment; 42% (34 cases) were symptomatic, the most frequent symptoms including: pain, vaginal hemorrhage and constitutional syndrome.

Relapse was detected mainly through symptoms and physical findings (54.3%), followed by increased marker levels (29.6%), computed tomography (CT) (9.9%), and abnormal vaginal cytology findings (6.2%). A total of 45 patients died due to the disease. Postrelapse global survival rates were 74% at 2 years, 40.5% at 5 years and 32.8% at 10 years. In the multivariate analysis, only histological grade G3 and LVSI in the initial biopsy were associated with lower global survival ( *p* < 0.007 and *p* < 0.002, respectively). No significant survival differences were found for single versus multiple relapse episodes ( *p* = 0.18) or for different relapse sites (local-regional, ganglionar or distant) ( *p* = 0.28). No differences were found in global survival between patients with symptomatic or asymptomatic relapse ( *p* = 0.7) ( Table 2 ).

**Table 2:** Multivariate survival analysis of patients with endometrial cancer relapse

Variables	HR	95%CI		*p-value*
Stage III-IV vs Stage I-II	1.02	0.499	2.129	0.9
Grade 1 vs Grade 2 Grade 1 vs Grade 3	5.002 4.310	1.539 1.458	16.255 12.741	0.007 0.008
Histologic type Type 1 Type 2	0.62	3.41	0.3	0.38
Lymphovascular space involvement	2.714	1.429	6.328	0.002
Type of relapse Local-regional Distant	1.180	0.709	1.984	0.28
Single relapse episode vs Multiple relapse episodes	1.85	0.87	3.94	0.18
Symptomatic vs Asymptomatic relapse	0.94	0.33	2.65	0.7

Abbreviations: CI, confidence interval; HR, Hazard ratio.

## Discussion

Currently, no controlled studies support patient follow-up after the termination of treatment or indicate that such follow-up can improve survival. 5 6 Several studies have demonstrated that the global rate of endometrial cancer relapse after a completed primary treatment is relatively low, ∼ 13% (95% confidence interval [CI]: 11–14%), with age, histological grade, mitotic activity, myometrial invasion, lymphovascular invasion and ganglionar involvement as main prognostic factors. 7 8 In our series, which included > 800 cases, we found a 10.4% relapse rate, in line with other published data.

In 2016, the European Society of Medical Oncology (ESMO), the European Society of Gynaecological Oncology (ESGO) and the European Society of Therapeutic Radiology Oncology (ESTRO) published a consensus document on the classification of the risk of relapse, creating new subgroups to help design better adjusted and tailored treatments. 9

In our study, 73.3% of recurrences belonged to the high-risk groups, and most relapse cases occurred within the 1 ^st^ 3 years of follow-up (80.2%), in agreement with data published by other authors. 5 10

Regarding the site of relapse, Morice at al. 11 showed in their series that distant metastases were more frequent than local relapse. Carraca et al., 12 in a retrospective study including 282 patients, found local disease in 40.6%, distant disease in 32.5%, and concomitant local plus distant relapse in 26.9%. In our study, we found 41.9% cases of distant relapse, which was in agreement with the available evidence.

The role of follow-up is based on the concept that detecting relapse while still asymptomatic allows for better therapeutic options and outcomes. However, even with intensive surveillance, relapse is frequently detected through symptoms, which occur in 41 to 83% of patients. 7 13

Sartori et al. 5 reported poorer outcomes in women with symptomatic relapse than in asymptomatic ones, diagnosed through clinical examination or imaging tests. Carrara et al. 12 reported higher median survival in asymptomatic than in symptomatic relapse (35 months versus 13 months) and concluded that early diagnosis, achieved through a planned follow-up procedure, enhanced clinical outcomes in these patients. However, other authors failed to detect differences in the survival of patients with asymptomatic versus symptomatic relapse. 6 14 Otsuka et al. 15 studied 51 patients with endometrial cancer relapse and found that the site of relapse and the time to relapse were independent prognostic factors influencing survival, although they also failed to find significant differences between patients with or without symptoms. They concluded that detecting asymptomatic relapse through imaging tests or tumor marker levels did not influence the prognosis.

In our series, 42% of relapse cases were symptomatic; and no significant differences were found in the survival of patients with versus without symptoms ( *p* = 0.7). Hence the importance of providing good information about symptom detection and advising patients to seek medical advice as soon as they appear.

In the present study, > 50% of the cases of relapse were detected through symptoms and physical examination; followed by elevated levels of tumor marker Ca 125 (29.6% of the cases) and imaging techniques (9.9% of the cases). In other studies, cytology of the vaginal vault detected between 0 and 6.8% relapse in asymptomatic patients, most of them in early-stage low-grade disease. 9 16 In our study, cytology detected 6.2% of the relapses in the absence of symptoms or of physical examination findings. Thus, medical surveillance through vaginal cytology was not particularly efficient in detecting endometrial cancer relapse. We propose that the systematic use of cytology as a part of follow-up should be discouraged. Hunn et al., 6 in a series of 92 cases of high-grade endometrial cancer relapse, showed that elevated Ca 125 levels detected 20% of asymptomatic relapses. Although routine measurement of CA-125 levels is not advised in patients at the initial stages of endometrial cancer, it may be suitable in selected patients with advanced disease, serous histology or elevated CA-125 levels before treatment. 10

The usefulness of imaging tests in endometrial cancer follow-up is not clear. Similarly to our findings, other studies reported that CT detected 5 to 20.8% of asymptomatic endometrial cancer relapses. 6 13 The usefulness of pelvic ultrasound in local relapse detection is under study; although its detection rate is between 4% and 31%, 10 namely lower than detection through physical examination. These findings indicate that imaging techniques should also not be recommended in routine follow-up of asymptomatic patients, but should only when they were clinically indicated.

There is little evidence that the current approach to monitoring endometrial cancer in a hospital setting has an impact on survival. Given the rising number of gynecologic cancer survivors, intensive oncology specialist follow-up might turn unsustainable; additionally, it may be unnecessary for many patients. 17 18 In this regard, two clinical trials aimed at evaluating the efficacy of different endometrial cancer follow-up schedules are in course: the TOTEM trial (NCT00916708) 19 and the ENSURE trial (NCT02413606). 20

There is growing evidence that cancer survivors have long-term physical, psychological and social needs that are not addressed in the traditional hospital follow-up. Thus, we postulate that the strategy of care should start to shift from a purely disease-focused to a more comprehensive health-focused approach. 21 22

The main limitation of the present study was its retrospective nature. However, it has several strong points, such as including a significant number of patients (despite being a single center study) and the fact that all patients were managed by the same multidisciplinary team using identical follow-up protocols.

## Conclusion

In summary, the recurrence rate of endometrial cancer in our environment is low (10.04%). Taking into account that 80% of the cases occur in the 1 ^st^ 3 years post-treatment and that symptom evaluation and clinical examination are the most effective follow-up methods, a change should be considered in the current model of hospital-based monitoring. However, since most available studies are retrospective, prospective clinical trials (like the above-mentioned ones) are important to determine the actual role of follow-up procedures in endometrial cancer.

## References

[1] Sociedad Española de Oncología Médica Las cifras del cáncer en España: Depósito Legal: M-3266–2020 [Internet] 2020[cited 2020 Jan 15]. Available from:https://seom.org/seomcms/images/stories/recursos/Cifras_del_cancer_2020.pdf

[2] Bray F Ferlay J Soerjomataram I Siegel R L Torre L A Jemal A Global cancer statistics 2018: GLOBOCAN estimates of incidence and mortality worldwide for 36 cancers in 185 countries CA Cancer J Clin 2018 68 06 394 424 10.3322/caac.21492 30207593

[3] Morrow C P Bundy B N Kurman R J Creasman W T Heller P Homesley H D Graham J E Relationship between surgical-pathological risk factors and outcome in clinical stage I and II carcinoma of the endometrium: a Gynecologic Oncology Group study Gynecol Oncol 1991 40 01 55 65 10.1016/0090-8258(91)90086-k 1989916

[4] Rubinstein E B Miller W L Hudson S V Howard J O'Malley D Tsui J Cancer Survivorship Care in Advanced Primary Care Practices: A Qualitative Study of Challenges and Opportunities JAMA Intern Med 2017 177 12 1726 1732 10.1001/jamainternmed.2017.4747 28973067 PMC5820731

[5] Sartori E Pasinetti B Chiudinelli F Gadducci A Landoni F Maggino T Surveillance procedures for patients treated for endometrial cancer: a review of the literature Int J Gynecol Cancer 2010 20 06 985 992 10.1111/IGC.0b013e3181e2abcc 20683406

[6] Hunn J Tenney M E Tergas A I Bishop E A Moore K Watkin W Patterns and utility of routine surveillance in high grade endometrial cancer Gynecol Oncol 2015 137 03 485 489 10.1016/j.ygyno.2015.03.047 25838164 PMC4484273

[7] Cancer Care Ontario Program in Evidence-based Care Gynecology Cancer Disease Site Group Fung-Kee-Fung M Dodge J Elit L Lukka H Chambers A Oliver T Follow-up after primary therapy for endometrial cancer: a systematic review Gynecol Oncol 2006 101 03 520 529 10.1016/j.ygyno.2006.02.011 16556457

[8] Iavazzo C Gkegkes I D Vrachnis N Early recurrence of early stage endometrioid endometrial carcinoma: possible etiologic pathways and management options Maturitas 2014 78 03 155 159 10.1016/j.maturitas.2014.04.009 24815295

[9] ESMO-ESGO-ESTRO Endometrial Consensus Conference Working Group Colombo N Creutzberg C Amant F Bosse T González-Martin A Ledermann J ESMO-ESGO-ESTRO Consensus Conference on Endometrial Cancer: diagnosis, treatment and follow-up Ann Oncol 2016 27 01 16 41 10.1093/annonc/mdv484 26634381

[10] Salani R Khanna N Frimer M Bristow R E Chen L M An update on post-treatment surveillance and diagnosis of recurrence in women with gynecologic malignancies: Society of Gynecologic Oncology (SGO) recommendations Gynecol Oncol 2017 146 01 3 10 10.1016/j.ygyno.2017.03.022 28372871

[11] Morice P Levy-Piedbois C Ajaj S Pautier P Haie-Meder C Lhomme C Value and cost evaluation of routine follow-up for patients with clinical stage I/II endometrial cancer Eur J Cancer 2001 37 08 985 990 10.1016/s0959-8049(01)00066-1 11334723

[12] Carrara L Gadducci A Landoni F Maggino T Scambia G Galletto L Could different follow-up modalities play a role in the diagnosis of asymptomatic endometrial cancer relapses?: an Italian multicentric retrospective analysis Int J Gynecol Cancer 2012 22 06 1013 1019 10.1097/IGC.0b013e31825ad3ee 22706226

[13] Gadducci A Cosio S Fanucchi A Cristofani R Genazzani A R An intensive follow-up does not change survival of patients with clinical stage I endometrial cancer Anticancer Res 2000 20(3B):1977 1984 10928137

[14] Sartori E Laface B Gadducci A Maggino T Zola P Landoni F Zanagnolo V Factors influencing survival in endometrial cancer relapsing patients: a Cooperation Task Force (CTF) study Int J Gynecol Cancer 2003 13 04 458 465 10.1046/j.1525-1438.2003.13328.x 12911722

[15] Otsuka I Uno M Wakabayashi A Kameda S Udagawa H Kubota T Predictive factors for prolonged survival in recurrent endometrial carcinoma: Implications for follow-up protocol Gynecol Oncol 2010 119 03 506 510 10.1016/j.ygyno.2010.08.013 20837356

[16] Novetsky A P Kuroki L M Massad L S Hagemann A R Thaker P H Powell M A The utility and management of vaginal cytology after treatment for endometrial cancer Obstet Gynecol 2013 121 01 129 135 10.1097/aog.0b013e31827499a9 23262937 PMC3864642

[17] Erikson C Salsberg E Forte G Bruinooge S Goldstein M Future supply and demand for oncologists : challenges to assuring access to oncology services J Oncol Pract 2007 3 02 79 86 10.1200/JOP.0723601 20859376 PMC2793740

[18] Rutledge T L Kano M Guest D Sussman A Kinney A Y Optimizing endometrial cancer follow-up and survivorship care for rural and other underserved women: Patient and provider perspectives Gynecol Oncol 2017 145 02 334 339 10.1016/j.ygyno.2017.03.009 28325583 PMC7416737

[19] Beaver K Williamson S Sutton C Hollingworth W Gardner A Allton B Comparing hospital and telephone follow-up for patients treated for stage-I endometrial cancer (ENDCAT trial): a randomised, multicentre, non-inferiority trial BJOG 2017 124 01 150 160 10.1111/1471-0528.14000 27062690

[20] Trial between two follow up regimens with different test intensity in endometrial cancer treated patients (TOTEM) [Internet] 2018[cited 2019 Dec 20]. Available from:https://clinicaltrials.gov/ct2/show/NCT00916708

[21] Ezendam N PM de Rooij B H Kruitwagen R FPM Creutzberg C L van Loon I Boll D ENdometrial cancer SURvivors' follow-up carE (ENSURE): Less is more? Evaluating patient satisfaction and cost-effectiveness of a reduced follow-up schedule: study protocol of a randomized controlled trial Trials 2018 19 01 227 10.1186/s13063-018-2611-x 29661218 PMC5902894

[22] Jones J M Ferguson S Edwards E Walton T McCurdy N Howell D Experiences of care delivery: endometrial cancer survivors at end of treatment Gynecol Oncol 2012 124 03 458 464 10.1016/j.ygyno.2011.10.037 22079362

